# Grapevine trunk diseases of cold-hardy varieties grown in Northern Midwest vineyards coincide with canker fungi and winter injury

**DOI:** 10.1371/journal.pone.0269555

**Published:** 2022-06-03

**Authors:** David H. DeKrey, Annie E. Klodd, Matthew D. Clark, Robert A. Blanchette

**Affiliations:** 1 Department of Plant Pathology, University of Minnesota, St. Paul, Minnesota, United States of America; 2 University of Minnesota Extension, Farmington, Minnesota, United States of America; 3 Department of Horticultural Science, University of Minnesota, St. Paul, Minnesota, United States of America; Universidade do Minho, PORTUGAL

## Abstract

Grapevine trunk diseases make up a disease complex associated with several vascular fungal pathogenic species. Surveys to characterize the composition of grapevine trunk diseases have been conducted for most major grape growing regions of the world. This study presents a similar survey characterizing the fungi associated with grapevine trunk diseases of cold-hardy interspecific hybrid grape varieties grown nearly exclusively in the atypical harsh winter climate of Northern Midwestern United states vineyards. From the 172 samples collected in 2019, 640 isolates obtained by culturing were identified by ITS sequencing and represent 420 sample-unique taxa. From the 420 representative taxa, opportunistic fungi of the order Diaporthales including species of *Cytospora* and *Diaporthe* were most frequently identified. Species of *Phaeoacremonium*, *Paraconiothyrium*, and *Cadophora* were also prevalent. In other milder Mediterranean growing climates, species of Xylariales and Botryosphaeriales are often frequently isolated but in this study they were isolated in small numbers. No Phaeomoniellales taxa were isolated. We discuss the possible compounding effects of winter injury, the pathogens isolated, and management strategies. Additionally, difficulties in researching and understanding the grapevine trunk disease complex are discussed.

## Introduction

**Fig 1:**
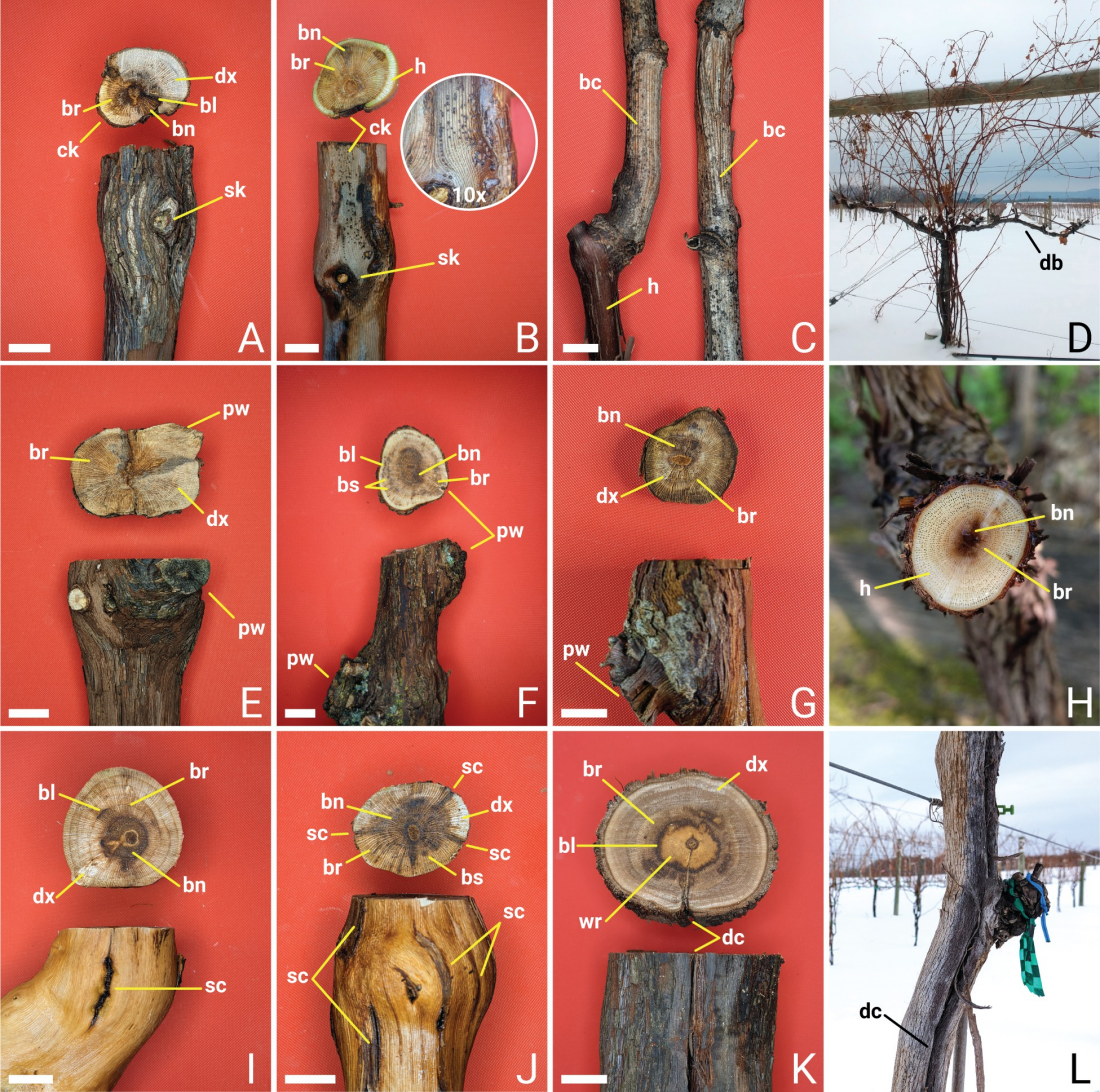
Symptoms of grapevine trunk diseases in Northern Midwest vineyards. Cankers were often associated with skips in the cordons but had rarely wedge-shaped discoloration (A). Cankers more often had irregular shaped xylem reactions (B). Pycnidia were sometimes observed fruiting from cankers (B) and bleached canes (C). Dieback symptoms are common and the result of successive skips starting from tips of cordons (D). Pruning wounds were associated with minor (E), moderate (F), and severe (G) vascular streaking symptoms. Near completely healthy vascular tissue observed in wild *Vitis riparia* vines (H). Infrequent shallow cracks (I), several shallow cracks (J), and deep cracks (K and L) were associated with minor to moderate (I), moderate to severe (J), and severe (K) vascular symptoms. Winter injury often results in deep cracks on the trunk (L). Observations included cankers (ck), skips in the cordons (sk), bleached canes (bc), dieback (db), pruning wounds (pw), shallow cracks (sc), deep cracks (dc), black spotting (bs), black lines (bl), brown-red wood streaking (br), brown to black necrotic streaking (bn), discolored xylem (dx), sometimes healthy tissue (h), and white rot (wr). Bars = 1 cm.

Grapevine trunk diseases (GTDs) make up a disease complex most often associated with several wood-inhabiting fungal species [1] and more recently possibly some bacterial species [2]. Sub-groups of these diseases are frequently categorized by symptomology and or taxonomic designation of causal fungal agents. Common names given to GTDs include Esca [3], folletage or berry shrivel [4], Petri disease, young esca, young vine decline [5], hoja de malvón [6], Botryosphaeria dieback, bot canker, black goo [7], slow stroke [8], eutypiosis, Eutypa dieback [9], black dead arm, dying arm, dead arm [10], swelling arm [11], grapevine leaf stripe disease [12], Phomopsis dieback, black spot [13], black measles [14], and black foot disease [15]. These diseases can be difficult to diagnose due to their sporadic symptom display and similarity of external and internal symptoms. Such symptoms may include interveinal foliar chlorosis and necrosis or tiger striping, generalized dieback, apoplexy or sudden death, gummosis, vascular streaking, wedge- or V-shaped vascular discoloration, cankers, and wood decay (Fig 1) [16].

GTDs cause serious grapevine health and economic problems and can be found in all grape growing regions of the world [17–19]. In the Northern Midwest United States (NMW), growers often struggle with unproductive cordon sections commonly referred to as “skips in the cordon” or “blind wood” (Fig 1A and 1B). Cordon skips come at the cost of vineyard managers with lower yields and require retraining new cordons. In the past, chemicals such as sodium arsenate was used to control GTDs but health [20] and environmental concerns [21] have eliminated its widespread use. Often the dramatic increase in the incidence of GTDs in the last two decades is associated with the 2003 ban on sodium arsenate [22]. However, the increasing incidence of GTDs in countries which have never used sodium arsenate points to other factors being involved [22]. To date, very few chemical options are available to growers but recent research in the use of biological control agents has shown some promise for controlling specific fungal GTD pathogens [23]. In most situations, development of best practices for GTD management remains the best option for growers. Management strategies can include practices such as variety selection, rootstock selection, training system, pruning timing, double-pruning, wound-protection, multi-trunking, trunk renewal, trunk surgery, debris removal, tool sterilization, and other practices [24]. Management options of GTD pathogens tend to be region specific with considerations to climate, weather, cultural practices, and varieties grown. In the NMW, wine grape growing is a relatively new industry that is increasing at a considerable pace. According to the 2016 University of Minnesota Extension vineyards and grapes status report, planted cold-hardy grapevine varieties increased from 5900 acres to 7580 acres from 2011 to 2015 [25]. However, Tuck et al. also reported an average decrease in yield of 3.5 to 3.2 tons per acre from 2011 to 2015 which indicates a need for better-informed, variety and region-specific GTD management practices. To accomplish this, it is important to identify the GTDs responsible for the problems.

Traditional European *Vitis vinifera* cultivars are not often grown in the NMW due to difficulties brought on by harsh winters and a short growing season. Instead, own-rooted cold-hardy interspecific hybrid grape (CIHG) varieties are widely and often exclusively grown in the region. The genetic contribution of the native riverbank grape (*V*. *riparia*) provide CIHG varieties developed in Minnesota their cold-hardiness (rated down to -30°C) [26, 27] and some resistance to endemic diseases and insect pests like phylloxera [28, 29]. Over the past four decades, the University of Minnesota has become a leader in the development of several CIHG wine and table grape varieties. The varieties most produced in the region include Marquette, Frontenac blanc, Frontenac, La Crescent, Petite Pearl, Brianna, and Frontenac Gris [30].

As many NMW vineyards are now reaching a decade in age since their first vines were planted, the characteristic cordon skip (Fig 1A and 1B) and dieback (Fig 1D) symptoms of GTDs have begun to appear. In addition, the compounding effect that GTDs and winter injury have on vines is becoming a major concern (Fig 1L). In many other parts of the world where grapes are grown, surveys have been conducted to characterize the region-specific composition of GTD pathogens. In Europe and nearby Mediterranean countries where GTDs were first reported, major causal agents include fungal species of the genera *Eutypa*, *Diplodia*, *Botryosphaeria*, and *Phaeomoniella* [19]. Similar fungal species have also been identified as major causal agents of GTDs in Australia, New Zealand [31], South Africa [32], China [33–35], and Chile [36, 37] as well as southern US and west coast US [38, 39]. Species of *Fomitiporia* are often the main white-rot pathogen found in older vines in most of these regions as well [19]. Species of *Phaeoacremonium* have been identified in grapevines and other woody hosts in several countries around the world [40]. Species of *Cadophora* are on occasion identified as well notably found in Canada [41]. Species of *Diaporthe* and *Cytospora* have also been identified in most of these regions though often to a lesser extent and usually in more humid growing regions [32].

However, no surveys have been conducted in the NMW or exclusively on CIHG cultivars. The objective of this study was to identify the major GTD species throughout the grape-growing regions of Minnesota and Wisconsin. Three hypotheses were explored in this study. First, NMW GTDs will have a regionally distinct composition compared to other grape-growing regions of the world given the harsh growing climate and CIHG varieties grown. Second, dieback symptoms and internal vascular streaking can be associated with pruning wounds and winter injury. Third, isolation frequency of fungal genera will significantly differ compared to sample variety, variety berry color, sample section type, and sample county origin.

## Methods

### Sample collection

**Fig 2:**
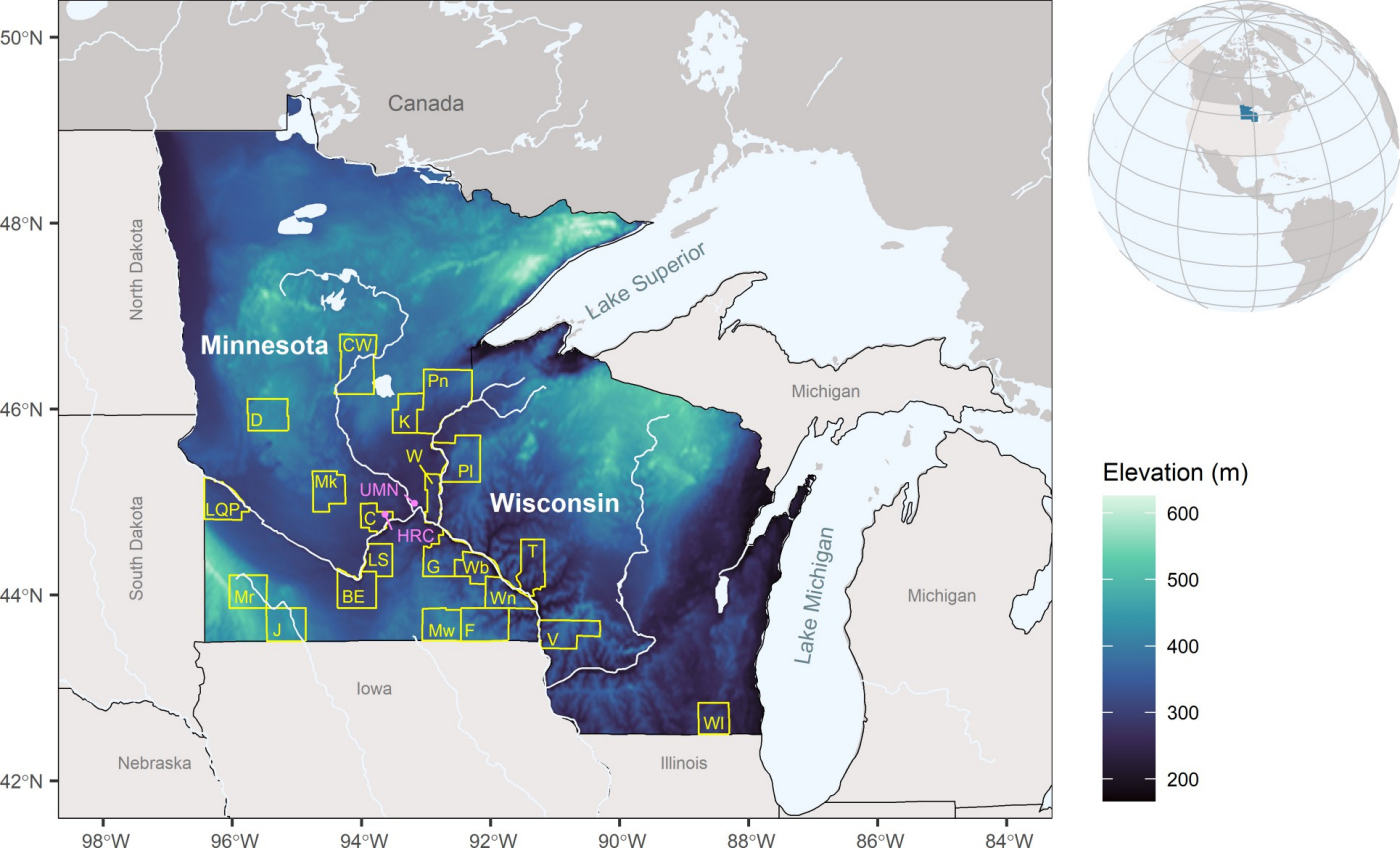
Map of counties sampled. Grapevine wood samples collected from Minnesota and Wisconsin counties traced in yellow included Blue Earth (BE), Carver (C), Crow Wing (CW), Douglas (D), Fillmore (F), Goodhue (G), Jackson (J), Kanabec (K), Lac Qui Parle (LQP), Le Sueur (LS), Meeker (Mk), Mower (Mw), Murray (Mr), Pine (Pn), Polk (Pl), Trempealeau (T), Vernon (V), Wabasha (Wb), Walworth (Wl), Washington (W), and Winona (Wn). Color scale indicates elevation in meters. Pink points denote locations of the University of Minnesota (UMN) St. Paul campus and the UMN Horticultural Research Center (HRC) where the grape breeding program is located and where several samples were collected. Map constructed in R with the public domain map collections Natural Earth (https://www.naturalearthdata.com/) and Terrain Tiles (https://registry.opendata.aws/terrain-tiles/).

Our sample collection was targeted towards symptomatic grapevines showing skips in the cordons, generalized dieback, reduced productivity, vascular discoloration, vascular decay, or apoplexy (Fig 1). A few externally asymptomatic vines were also collected for comparison. In 2019, a total of 172 samples were collected and brought to the laboratory. Samples were collected throughout both the dormant and growing season of 2019. Some samples were shipped by priority mail. Most samples collected were woody sections of grapevines, especially of cordons and trunks. It is important to note that regular re-trunking is frequently practiced in NMW vineyards and therefore main woody trunks of vines rarely, if ever, exceed ten years in age. Samples were stored at -20°C until processed. Samples were acquired from 34 vineyards in Minnesota and Wisconsin from a total of 21 counties (Fig 2). However, data reported in this study is down to the county level to conserve anonymity of contributing vineyards. Primarily named CIHG varieties were collected as well as a few wild vines and genetically unique breeding lines.

### Sample processing

Large diameter vine samples were cut and 3–5 mm^3^ chips excised from the margins of discolored or decayed internal vascular wood tissue or from the edge of cankers. For smaller diameter vine samples, the bark was peeled off and 3–5 mm thick discs were cut. Some disks were kept whole while others cut in half or in fourths depending on diameter of the sample. Excised chips where surface sterilized for 30 sec in an aqueous 10% sodium hypochlorite solution, followed by two washes in sterile distilled H_2_O, and one wash in 70% EtOH then left to dry in a clean air cabinet prior to plating. Between 3–5 chips per plate were then semi-embedded into 3 different culturing medias including malt extract agar (MEA; 15 g of Difco Bacto-agar, 15 g of Difco Bacto malt extract, and 1 L of deionized water with 0.1 g streptomycin sulphate dissolved in a small amount of 95% EtOH added post-autoclaving once cooled to 50°C), basidiomycete semi-selective agar (BSA; same as MEA recipe plus 2 g of Difco yeast extract and 0.06 g Aldrich benomyl dissolved in a small amount of 95% EtOH added pre-autoclaving with 2 mL 85% lactic acid added post-autoclaving once cooled to 50°C, adapted from Worrall, 1991) [42], and sabouraud dextrose agar (SDA; same as MEA recipe with 0.1 g Aldrich cycloheximide dissolved in a small amount of deionized water added post-autoclaving once cooled to 50°C, adapted from Harrington, 1981) [43]. Plates were left to incubate at 20–23°C in darkness and checked daily. Emerging fungi were transferred onto fresh MEA. All cultures were maintained and stored in plastic bins at 20–23°C.

### Isolate selection

Fungal isolates for each sample were selected by culture macro-morphology on MEA and genetic identification by sequencing the internal transcribed spacer (ITS) genomic region. The primary macro-morphological characteristics considered included isolate color, growth rate, hyphal branching, hyphal depth, hyphal extension, hyphal margin, fruiting, sporulation, and metabolite staining of media. At the start of this study all unidentified cultures with unique morphologies isolated from a single sample were selected for sequencing. Isolates were later selected by macro-morphology in a more targeted manner as the study progressed by choosing unique cultures or cultures similar to known pathogens we previously identified in the isolate collection. Any isolates with questionable, non-descript, or similar culture macro-morphology were sequenced to be sure of their identity.

### DNA extraction and amplification

The DNA of select isolates was extracted using the NaOH protocol according to Osmundson et al. (2013) [44]. Hyphae were scraped using a sterile scalpel from cultures of select isolates on MEA that had grown out larger than 2.5 cm in diameter. Hyphal tissue was transferred to a 1.7 mL microcentrifuge tube with 300 μL of 5 mM NaOH and 3 to 5 3.5 mm glass beads. The samples where then vortexed for 1 to 5 min and centrifuged for 30 sec at 10,000 rpm. Then 5 μL of supernatant was transferred to new tubes containing 495 μL Tris-HCL 5 mM, pH 8.0.

The ITS region of the isolated DNA was targeted for PCR amplification using the ITS1F/4 primer pair [45] according to Blanchette et al. (2016) [46]. Each PCR had a final volume of 25.5 μL consisting of 12.5 μL GoTaq® Green Master Mix, 9.5 μL molecular grade water, 1 μL of each primer at 10 μM, and 0.5 μL bovine serum albumin. The ITS locus was amplified using a Bio-Rad T100™ Thermal Cycler following a program of 94°C for 5 min, 35 cycles of 94°C for 1 min, 50°C for 1 min, and 72°C for 1 min, followed by a final extension step of 72°C for 5 min. Locus amplification was confirmed by gel electrophoresis of SYBR stained PCR products prior to sequencing. Crude PCR products were Sanger sequenced by ABI 3730xl DNA sequences, Applied Biosystems, Foster City, CA.

### Molecular identification

Sequences were processed using Geneious v9.0. The processed sequences where then identified with the basic local alignment search tool algorithm program for nucleotide sequences (BLASTn) initially against the TrunkDiseaseID.org [47] database and also against the standard complete NCBI GenBank. Best sequence identity match was selected for by consideration of highest score for published data as denoted in GenBank at the time of BLASTn searches. Identity of isolates were matched to published sequences from taxonomic studies and identified to the species level whenever possible. Isolates with greater than 97% sequences identity match were considered homologous. Pathogenicity of identified fungal species on grapevines were denoted according initially to TrunkDiseaseID.org [47] and then confirmed and expanded by an assortment of grapevine pathogenicity trials found in published literature. However, most pathogenicity trials for these fungi were conducted on traditional *V*. *vinifera* grapevine cultivars. Samples were scored as GTD+ upon sequence confirmation of at least one known pathogenic species. Additional isolation and sequencing was discontinued once a sample was designated GTD+.

### Data analysis

Data were analyzed using the R statistical programming language in the RStudio integrated development environment using an assortment of packages but most notably the collection of Tidyverse Packages (v1.3.0) [48], the iNEXT package (v2.0.20) [49] to analyze sample coverage, and the vcd package (v1.4–9) for multivariant analysis. The vcd package was used to explore potential differences in isolation frequencies of genera-level taxa compared to a few variables of interest that are descriptive of the 168 collected woody samples. In brief, Hill numbers are used in the iNEXT package to estimate and then visualized sample completeness [50]. Additionally, diversity Pearson residuals statistics were used to analyze the measure of discrepancy between observed and expected values within the vcd package. For each statistical comparison, a p-value is returned from a corresponding Chi-square test and a residual shaded mosaic plot was produced. Mosaic plots are graphs used for visualizing the comparison of multi-categorical data where both the x- and y-axis are sized proportionally to the input data, i.e. the sum area of the blocks represent 100% of the data and individual blocks are size proportionally to the frequency with which the categories are observed.

## Results

Internal symptoms of GTDs following the terminology of Mugnai et al. 1999 [16] included brown-red wood streaking in a clearly defined wedge-shape from the cambium to pith which is indicative of canker fungi were observed in few of our samples. Cankers more often occurred in irregular forms and were associated with skips in the cordons (Fig 1A and 1B). Centrally diffuse brown-red wood as well as brown to black necrotic streaking originating from the pith was often associated with pruning wounds (Fig 1E–1G) and cracks (Fig 1I–1L). All samples collected had discolored xylem to some extent and nearly completely healthy vascular tissues were only observed in some cross-sections of wild riverbank grapevines in forest and urban environments not included in this survey (Fig 1H). Cross-sections near pruning spurs often showed discolored wood symptoms without being preceded by diffuse brown-red wood or brown to black streaking (Fig 1E). Concentric black spotting, the result of longitudinal streaking, was also frequently observed, and in some cases, black spotting would begin to coalesce into shorter black lines (Fig 1F). Most samples had severe mottled expression of vascular symptoms especially for brown-red wood streaking, brown to black necrotic streaking, and discolored xylem (Fig 1G). Severe symptoms were sometimes associated with several points of origin from shallow cracking from winter injury or hail damage (Fig 1J). Rarely, if ever, have GTD foliar symptoms been observed in the NMW which possibly may be the result of our overall young vineyards or different climate. Foliar symptoms are more often observed in older vines under particular seasonal conditions [51, 52]. Moreover, lack of foliar symptoms may also be the result of regular re-trunking, a common cultural practice in the NMW. It is uncommon in the NMW for grapevine trunk wood to exceed ten years in age.

**Fig 3:**
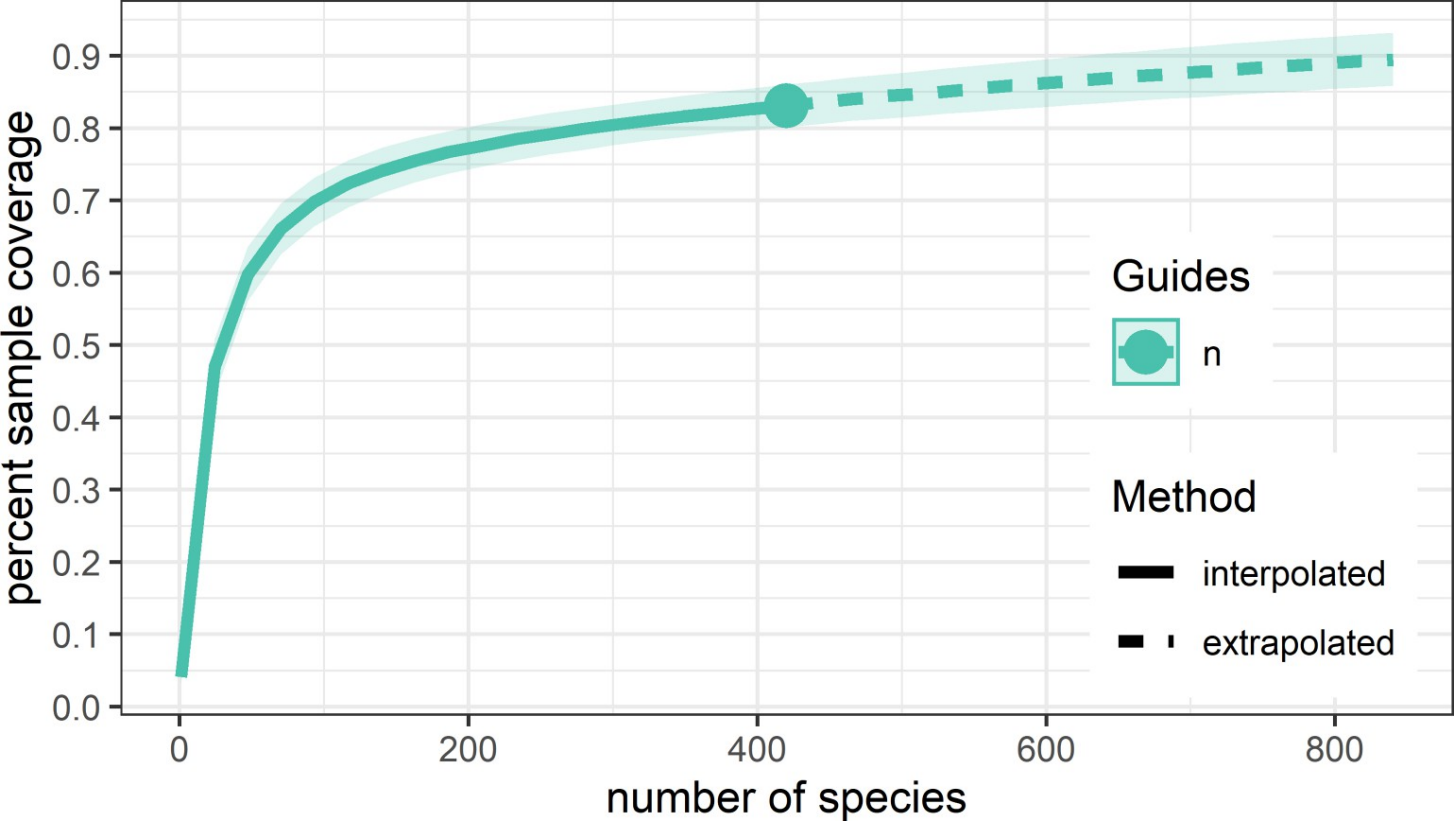
Rarefaction sample coverage curve. Observed sample coverage reaches 83% for the 420 sample representative taxa. Extrapolated sample coverage reaches 90% by doubling the number of sample representative taxa.

All wood samples collected had some degree of internal vascular symptoms including externally healthy samples (Fig 1). From 172 samples with various symptoms that included cankers and vascular discoloration, dieback as well as pruning wounds and cracks from cold injury or other environmental stresses yielded 640 isolates. These isolates represented 420 species-level taxa unique to individual samples. Rarefication using the 420 representative taxa estimate a sample coverage of 83% that reached to 90% by doubling the number of representative taxa (Fig 3).

We found 32 of the 34 sampled vineyard locations, 20 of 21 counties, in this survey to have at least one GTD+ sample. Of the 172 samples we collected, 142 (83%) had taxa reported as pathogens associated with GTDs. Most samples were cordon sections and of the Marquette variety (Tables 1 and 2). Most taxa are of the phylum Ascomycota (398 isolates, 94.76%) which encompass 19 different orders, 38 genera, and potentially 89 species (Table 3).

**Table 1:** Type of samples collected from the Northern Midwest in 2019. The majority of the 172 samples included woody sections of cordon, trunk, root, sucker, shoots, and unknown.

Type	Sample no.	Percent
cordon	113	66%
trunk	32	19%
root	11	6%
sucker	5	3%
shoot	4	2%
unknown	3	2%
slime flux	2	1%
bark	1	1%
basidiocarp	1	1%

**Table 2:** Varieties of the 172 samples collected in the Northern Midwest in 2019.

Variety	Sample no.	Percent
Marquette	42	24%
La Crescent	28	16%
Frontenac	20	12%
St. Pepin	9	5%
Frontenac Blanc	8	5%
Brianna	7	4%
Frontenac Gris	7	4%
Edelweiss	6	3%
Itasca	6	3%
Marechal Foch	6	3%
unknown	6	3%
Petite Pearl	3	2%
Prairie Star	3	2%
Valiant	3	2%
MN1069	2	1%
MN1016	2	1%
Sabrevois	2	1%
slime flux	2	1%
St. Croix	2	1%
MN43765	1	1%
basidocarp	1	1%
Millot	1	1%
MN1005	1	1%
Osceola Muscat	1	1%
Riverbank Grape	1	1%
Sauvignon	1	1%
Virginia Creeper	1	1%

**Fig 4:**
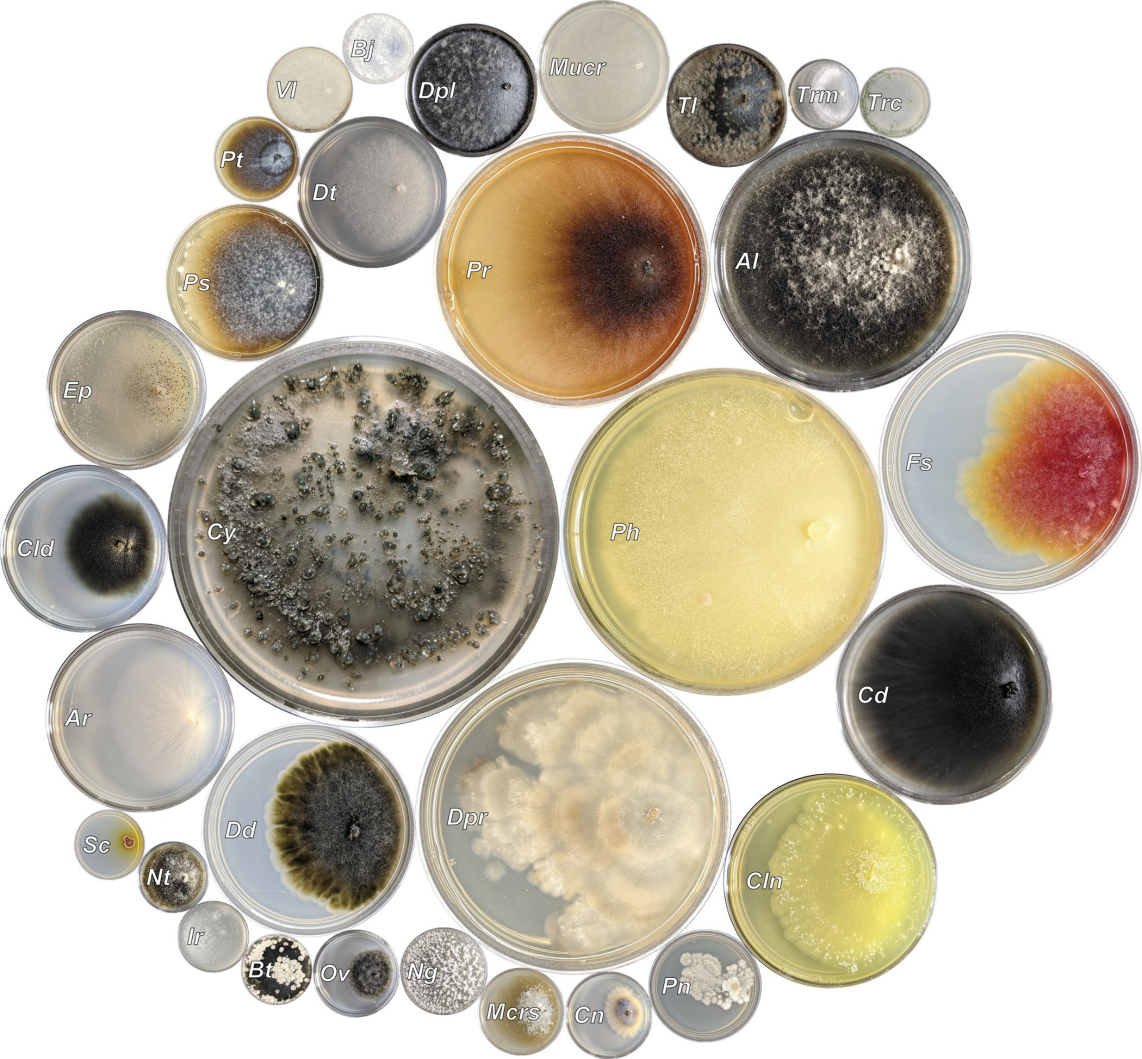
Fungal genera isolated from more than one grapevine sample. Individual culture image areas are relatively proportional to the number of samples each fungal genus was isolated from. All pictured fungal isolates grown on MEA. *Cytospora* (*Cy* = 60, GI-174, 80 dpi); *Phaeoacremonium* (*Ph* = 41, GI-422, 94 dpi); *Diaporthe* (*Dpr* = 38, GI-449, 47 dpi); *Paraconiothyrium* (*Pr* = 30, GI-516, 47 dpi); *Alternaria* (*Al* = 27, GI-879, 36 dpi); *Fusarium* (*Fs* = 27, GI-151, 17 dpi); *Clonostachys* (*Cln* = 18, GI-870, 27 dpi); *Cadophora* (*Cd* = 16, GI-370, 94 dpi); *Didymella* (*Dd* = 16, GI-257, 21 dpi); *Aureobasidium* (*Ar* = 14, GI-886, 21 dpi); *Cladosporium* (*Cld* = 11, GI-866, 21 dpi); *Epicoccum* (*Ep* = 10, GI-837, 19 dpi); *Pestalotiopsis* (*Ps* = 10, GI-491, 40 dpi); *Diatrypella* (*Dt* = 9, GI-464, 15 dpi); *Diplodia* (*Dpl* = 7, GI-408, 20 dpi); *Mucor* (*Mucr* = 7, GI-71, 20 dpi); *Talaromyces* (*Tl* = 6, GI-801, 55 dpi); *Penicillium* (*Pn* = 5, GI-337, 14 dpi); *Coniothyrium* (*Cn* = 3, GI-882, 21 dpi); *Microsphaeropsis* (*Mcrs* = 3, GI-505, 20 dpi); *Nigrospora* (*Ng* = 3, GI-846, 14 dpi); *Ovatospora* (*Ov* = 3, GI-828, 19 dpi); *Pithomyces* (*Pt* = 3, GI-162, 28 dpi); *Valsa* (*Vl* = 3, GI-384, 35 dpi); *Bjerkandera* (*Bj* = 2, GI-417, 28 dpi); *Botryosphaeria* (*Bt* = 2, GI-225, 35 dpi); *Irpex* (*Ir* = 2, GI-806, 55 dpi); *Nothophoma* (*Nt* = 2, GI-878, 31 dpi); *Sclerostagonospora* (*Sc* = 2, GI-140, 17 dpi); *Trametes* (*Trm* = 2, GI-68, 9 dpi); and *Trichoderma* (*Trc* = 2, GI-795, 14 dpi).

The most frequently isolated genera obtained in this study that were known to be associated with GTDs from previous reports included *Cytospora*, *Phaeoacremonium*, *Diaporthe*, *Cadophora*, *Pestalotiopsis*, *Diatrypella*, *Diplodia*, and *Botryosphaeria*, respectively (Fig 4). Of these genera the most frequent species level sequence matches associated with GTDs included *Cy*. *viticola*, *Ph*. *fraxinopennsylvanicum*, *Ph*. *minimum*, *Dpr*. *ampelina*, *Cd*. *luteo-olivacea*, *Ps*. *neglecta*, *Dt*. *verruciformis*, *Dpl*. *seriata*, and *Bt*. *dothidea* (Table 3).

**Table 3:** Taxonomy, isolation frequency, and pathogenicity of fungi identified by ITS with greater than 97% homology match.

Phylum (n)	Family (n)	Genus (n)	Isolate	Host variety	Origin	GenBank
Order (n)		Species (n) [pathogenicity studies]				
**Ascomycota (398)**						
Diaporthales (98)	Valsaceae (61)	*Cytospora* (60)	** **	** **	** **	** **
		*Cy*. *viticola* (57) [53]	GI-174	Frontenac Gris	Blue Earth, MN	OM307727
		*Cy*. *piceae* (2)	GI-847	Virginia Creeper	Carver, MN	OM307728
		Cy. mali (1)	GI-89	Marquette	Meeker, MN	OM307729
		*Valsa* (3)				
		*Vl*. *sordida* (2)	GI-384	Frontenac	Crow Wing, MN	OM307730
		*Vl*. *salicina* (1)	GI-413	Frontenac Gris	Douglas, MN	OM307731
	Diaporthaceae (37)	*Diaporthe* (38)				
		*Dpr*. *ampelina* (31) [54–56]	GI-856	Frontenac	Carver, MN	OM307732
		*Dpr*. *eres* (7) [54]	GI-236	Marquette	Goodhue, MN	OM307733
Togniniales (40)	Togniniaceae (40)	*Phaeoacremonium* (41)				
		*Ph*. *fraxinopennsylvanicum* (22) [41]	GI-347	St. Pepin	Goodhue, MN	OM307734
		*Ph*. *minimum* (15) [3, 38]	GI-422	Marquette	Douglas, MN	OM307735
		*Ph*. *amstelodamense* (1)	GI-212	Edelweiss	Goodhue, MN	OM307736
		*Ph*. *angustius* (1) [57]	GI-741	La Crescent	Carver, MN	OM307737
		*Ph*. *canadense* (1) [41]	GI-31	St. Pepin	Pine, MN	OM307738
		*Ph*. *hungaricum* (1)	GI-75	Osceola Muscat	Wabasha, MN	OM307739
Pleosporales (99)	Didymosphaeriaceae (31)	*Paraconiothyrium brasiliense* (30) [58, 59]	GI-516	Frontenac	Douglas, MN	OM307740
		*Paraphaeosphaeria sporulosa* (1)	GI-157	Edelweiss	Wabasha, MN	OM307741
	Didymellaceae (28)	*Didymella* (16)				
		*Dd*. *pinodella* (11)	GI-257	Marquette	Wabasha, MN	OM307742
		*Dd*. *glomerata* (2)	GI-116	MN43765	Carver, MN	OM307743
		*Dd*. *pomorum* (2)	GI-787	MN1016	Carver, MN	OM307744
		*Dd*. *bellidis* (1)	GI-389	La Crescent	Walworth, WI	OM307745
		*Epicoccum* (10)				
		*Ep*. *nigrum* (9)	GI-837	La Crescent	Carver, MN	OM307746
		*Ep*. *sorghinum* (1)	GI-223	Marquette	Blue Earth, MN	OM307747
		*Nothophoma spiraeae* (2)	GI-878	Riverbank Grape	Carver, MN	OM307748
	Pleosporaceae (26)	*Alternaria* (26)				
		*Al*. *alternata* (19)	GI-879	La Crescent	Carver, MN	OM307749
		*Al*. *tenuissima* (5)	GI-885	La Crescent	Blue Earth, MN	OM307750
		*Al*. *arborescens* (1)	GI-190	Frontenac	Wabasha, MN	OM307751
		*Al*. *infectoria* (1)	GI-829	Marquette	Blue Earth, MN	OM307752
	Astrosphaeriellaceae (3)	*Pithomyces chartarum* (3)	GI-162	Frontenac Gris	Blue Earth, MN	OM307753
	Coniothyriaceae (3)	Coniothyrium palmicola (3)	GI-882	La Crescent	Blue Earth, MN	OM307754
	Phaeosphaeriaceae (3)	*Sclerostagonospora* (2)				
		*Sc*. *cycadis* (1)	GI-141	Marquette	Meeker, MN	OM307755
		*Sc*. *lathyri* (1)	GI-140	Marquette	Blue Earth, MN	OM307756
		*Neosetophoma cerealis* (1)	GI-480	Frontenac Blanc	Crow Wing, MN	OM307757
	undefined family (3)	*Microsphaeropsis olivacea* (3) [60]	GI-505	Marquette	Lac Qui Parle, MN	OM307758
	Cucurbitariaceae (1)	*Neocucurbitaria quercina* (1)	GI-37	Marquette	Meeker, MN	OM307759
Hypocreales (49)	Nectriaceae (29)	*Fusarium* (26)				
		*Fs*. *acuminatum* (5)	GI-820	Frontenac	Fillmore, MN	OM307760
		*Fs*. *equiseti* (3)	GI-95	MN1005	Carver, MN	OM307761
		*Fs*. *solani* (3)	GI-376	Frontenac	Crow Wing, MN	OM307762
		*Fs*. *culmorum* (1)	GI-151	La Crescent	Carver, MN	OM307763
		*Ilyonectria liriodendri* (1) [61–63]	GI-322	Marechal Foch	Goodhue, MN	OM307764
		*Scolecofusarium ciliatum* (1)	GI-149	La Crescent	Carver, MN	OM307765
		*Thyronectria austroamericana* (1)	GI-796	slime flux	Carver, MN	OM307766
	Bionectriaceae (17)	*Clonostachys* (18)				
		*Cln*. *rosea* (16) [60]	GI-870	La Crescent	Polk, MN	OM307767
		*Cln*. *byssicola* (1)	GI-874	Marquette	Trempealeau, WI	OM307768
	Hypocreaceae (2)	*Trichoderma* (2)				
		*Trc*. *atroviride* (1)	GI-795	slime flux	Carver, MN	OM307769
		*Trc*. *deliquescens* (1)	GI-351	Frontenac Blanc	Fillmore, MN	OM307770
Xylariales (25)	Sporocadaceae (11)	*Pestalotiopsis* (10)				
		*Ps*. *neglecta* (6)	GI-491	Edelweiss	Trempealeau, WI	OM307771
		*Ps*. *uvicola* (2) [38, 56]	GI-738	Edelweiss	Trempealeau, WI	OM307772
		*Ps*. *brassicae* (1)	GI-403	Marquette	Lac Qui Parle, MN	OM307773
		*Ps*. *chamaeropis* (1)	GI-231	St. Pepin	Goodhue, MN	OM307774
		*Neopestalotiopsis mesopotamica* (1)	GI-220	Frontenac Gris	Blue Earth, MN	OM307775
		*Seimatosporium lichenicola* (1)	GI-99	Marquette	Carver, MN	OM307776
		*Seiridium rosarum* (1)	GI-352	La Crescent	Murray, MN	OM307777
	Diatrypaceae (9)	*Diatrypella*				
		*Dt*. *verruciformis* (8) [64, 65]	GI-464	Marquette	Carver, MN	OM307778
		*Dt*. *pulvinata* (1)	GI-416	Valiant	Douglas, MN	OM307779
		*Diatrype stigma* (1) [65]	GI-895	Valiant	Crow Wing, MN	OM307780
	Hypoxylaceae (2)	*Hypomontagnella submonticulosa* (1)	GI-817	Frontenac Blanc	Blue Earth, MN	OM307781
		*Hypoxylon invadens* (1)	GI-350	La Crescent	Blue Earth, MN	OM307782
	Apiosporaceae (1)	*Arthrinium arundinis* (1)	GI-70	Frontenac	Carver, MN	OM307783
	Xylariaceae (2)	*Rosellinia corticium* (1)	GI-62	St. Croix	Wabasha, MN	OM307784
Helotiales (21)	undefined family (18)	*Cadophora* (16)				
		*Cd*. *luteo-olivacea* (13) [41, 66–68]	GI-370	La Crescent	Mower, MN	OM307785
		*Cd*. *melinii* (2) [68]	GI-316	Marechal Foch	Goodhue, MN	OM307786
		*Cd*. *ferruginea* (1)	GI-328	La Crescent	Goodhue, MN	OM307787
	Dermateaceae (1)	*Discohainesia oenotherae* (1)	GI-442	Frontenac Gris	Winona, MN	OM307788
	Porodiplodiaceae (1)	*Porodiplodia vitis* (1)	GI-269	Frontenac	Blue Earth, MN	OM307789
	Sclerotiniaceae (1)	*Botrytis cinerea* (1)	GI-386	Frontenac	Walworth, WI	OM307790
Dothideales (14)	Saccotheciaceae (14)	*Aureobasidium pullulans* (14)	GI-886	La Crescent	Blue Earth, MN	OM307791
Botryosphaeriales (10)	Botryosphaeriaceae (10)	*Diplodia* (7)				
		*Dpl*. *seriata* (6) [38, 56, 58, 64, 69]	GI-408	La Crescent	Blue Earth, MN	OM307792
		*Dpl*. *corticola* (1) [38, 56]	GI-373	Petite Pearl	Crow Wing, MN	OM307793
		*Botryosphaeria dothidea* (2) [38, 56, 70]	GI-225	Marquette	Blue Earth, MN	OM307794
		*Phaeobotryon negundinis* (1)	GI-131	MN1005	Carver, MN	OM307795
Cladosporiales (11)	Cladosporiaceae (11)	*Cladosporium* (11)				
		*Cld*. *cladosporioides* (9)	GI-866	Marquette	Blue Earth, MN	OM307796
		*Cld*. *anthropophilum* (1)	GI-209	Marquette	Blue Earth, MN	OM307797
		*Cld*. *westerdijkiae* (1)	GI-194	Marquette	Blue Earth, MN	OM307798
	Trichocomaceae (4)	*Talaromyces amestolkiae* (4)	GI-801	*Vitis* spp.	Carver, MN	OM307799
Eurotiales (8)	Aspergillaceae (4)	*Penicillium* (5)				
		*Pn*. *pulvillorum* (2)	GI-337	Marquette	Blue Earth, MN	OM307800
		*Pn*. *raistrickii* (1)	GI-309	St. Pepin	Goodhue, MN	OM307801
		*Pn*. *simplicissimum* (1)	GI-210	Marquette	Blue Earth, MN	OM307802
		*Pn*. *sumatraense* (1)	GI-36	Marquette	Meeker, MN	OM307803
Sordariales (6)	Chaetomiaceae (4)	*Ovatospora* (3)				
		*Ov*. *brasiliensis* (2)	GI-828	Marquette	Blue Earth, MN	OM307804
		*Ov*. *mollicella* (1)	GI-881	La Crescent	Blue Earth, MN	OM307805
		*Chaetomium concavisporum* (1)	GI-865	Marquette	Blue Earth, MN	OM307806
	Sordariaceae (1)	*Sordaria fimicola* (1)	GI-848	Virginia Creeper	Carver, MN	OM307807
Trichosphaeriales (3)	Trichosphaeriaceae (3)	*Nigrospora oryzae* (3)	GI-846	Edelweiss	Winona, MN	OM307808
Chaetomellales (1)	Chaetomellaceae (1)	*Chaetomella raphigera* (1)	GI-217	Marquette	Goodhue, MN	OM307809
Chaetothyriales (1)	Herpotrichiellaceae (1)	*Rhinocladiella quercus* (1)	GI-486	Marquette	Blue Earth, MN	OM307810
Coniochaetales (1)	Coniochaetaceae (1)	*Coniochaeta velutina* (1)	GI-472	Brianna	Winona, MN	OM307811
Glomerellales (1)	Glomerellaceae (1)	*Colletotrichum acutatum* (1)	GI-287	La Crescent	Goodhue, MN	OM307812
Saccharomycetales (1)	Dipodascaceae (1)	*Geotrichum candidum* (1)	GI-164	Edelweiss	Goodhue, MN	OM307813
Thelebolales (1)	Thelebolaceae (1)	*Thelebolus microsporus* (1)	GI-88	Frontenac Blanc	Pine, MN	OM307814
Valsariales (1)	Valsariaceae (1)	*Valsaria spartii* (1)	GI-43	Marquette	Meeker, MN	OM307815
**Basidiomycota (15)**						
Polyporales (9)	Phanerochaetaceae (3)	*Bjerkandera adusta* (2)	GI-417	Marquette	Trempealeau, WI	OM307816
		*Hyphodermella rosae* (1)	GI-823	Frontenac	Fillmore, MN	OM307817
	Irpicaceae (2)	*Irpex lacteus* (2)	GI-806	*Vitis* spp.	Carver, MN	OM307818
	Polyporaceae (2)	*Trametes versicolor* (2)	GI-68	La Crescent	Wabasha, MN	OM307819
	Cerrenaceae (1)	*Cerrena unicolor* (1)	GI-198	Prairie Star	Carver, MN	OM307820
	Meruliaceae (1)	*Phlebia radiata* (1)	GI-798	*Vitis* spp.	Carver, MN	OM307821
Russulales (2)	Peniophoraceae (1)	*Peniophora cinerea* (1)	GI-342	La Crescent	Blue Earth, MN	OM307822
	Stereaceae (1)	*Stereum complicatum* (1)	GI-200	Marquette	Goodhue, MN	OM307823
Agaricales (2)	Physalacriaceae (1)	*Cylindrobasidium laeve* (1)	GI-263	Itasca	Wabasha, MN	OM307824
	Schizophyllaceae (1)	*Chondrostereum purpureum* (1)	GI-444	Itasca	Le Sueur, MN	OM307825
Hymenochaetales (1)	Hymenochaetaceae (1)	*Phellinus conchatus* (1)	GI-805	*Vitis* spp.	Carver, MN	OM307826
Cystofilobasidales (1)	Mrakiaceae (1)	*Tausonia pullulans* (1)	GI-61	slime flux	Carver, MN	OM307827
**Mucoromycota (7)**						
Mucorales (7)	Mucoraceae (7)	*Mucor* (7)				
		*Mucr*. *circinelloides* (6)	GI-71	La Crescent	Vernon, WI	OM307828
		*Mucr*. *moelleri* (1)	GI-365	Marquette	Mower, MN	OM307829

Taxonomic rankings from order to species are denoted followed by isolation frequency in parenthesis. The isolation frequency is the count of samples each taxa was isolated from a possible 172 samples. Representative isolates deposited to GenBank are listed for each species along with the sample variety and county origin of that isolate. Pathogenicity studies conducted for each species are listed in brackets following species. See references for complete citations. Highlighted species have associated pathogenicity studies. Highlighted isolates pictured in Fig 4.

There were 15 taxa of Basidiomycota (3.57%) which encompass 5 orders, 11 genera, and 12 species. *Bjerkandera adusta*, *Irpex lacteus*, and *Trametes versicolor* where the most frequently identified Basidiomycota isolated. These fungi were present in 5 samples but were found in counties not adjacent to one another. Very few taxa of Mucoromycota (7 isolates, 1.68%) were identified. *Mucor circinelloides*, not considered associated with GTD, was isolated from 6 samples which originated from different vineyards in nonadjacent counties.

There were no significant differences in isolation frequencies of genera based on sample berry color (S1 Fig). There is some indication of significant isolation frequency differences of genera by sample variety (S2 Fig) or sample county origin (S3 Fig). For most genera not enough sample representative taxa were obtained to be sure these isolation frequency differences between counties are truly significant. However, *Cadophora* obtained in this study notably had significant differences in isolation frequency between sample section types with a p-value of 0.013. *Cadophora* spp. were less often isolated from cordons with a Pearson residual of -2.9 and more frequently isolated from root, trunk, and sucker sections with Pearson residuals between 2.2 to 3.9 (S4 Fig).

## Discussion

The vast majority of the fungal taxa isolated in this study are of the phylum Ascomycota, several of which are considered pathogenic to grapevines (references cited in Table 3). *Cytospora* spp. and *Diaporthe* spp. of the order Diaporthales as well as *Phaeoacremonium* spp. make up the largest majority of isolates identified and are known to be pathogenic on grapevines. Frequent species identification of *Phaeoacremonium* in this study aligns similarly with most other GTD surveys. However, the other major results confirm our first hypothesis that the composition of GTDs for the NMW is different in comparison to most other studied grape growing regions. These fungi in the Diaporthales typically are considered a minor or secondary group of causal agents in other regions were GTD surveys were completed [55, 71–74] but in the study presented here they are the most commonly found GTD fungi in the NMW. The GTD species genera of *Diaporthe* was previously named *Phomopsis* by some other investigators [55, 75]. Species of *Cytospora* have been reported previously as prevalent in cold climate regions [74] and areas of high humidity [32].

The etiology of these Diaporthales pathogens on grapevines has been previously well characterized [76]. In addition to symptoms of internal wood discoloration, pathogenic species of *Cytospora* and *Diaporthe* can induce symptoms of cane bleaching (Fig 1C). They also produce asexual fruiting bodies known as pycnidia which can be found on all affected tissues (Figs 1B, 1C and 4). On succulent green tissue, pycnidia may be surrounded by a halo or half halo of chlorotic tissues or may reside hidden just under the bark of infected vines. Conidio-spores that ooze out from pycnidia serve as a major source of inocula that can re-infect the same host or infect other nearby hosts. Conidia are most often disseminated by rain or irrigation splash but also by contaminated tools and more rarely by wind alone. These species overwinter in colonized wood of canes, spurs, pruning debris, and dormant buds [77]. However, symptoms appear to differ from locations of sample collection and isolation particularly for *Diaporthe* spp. [11, 55, 78, 79]. Symptom differences may be explained by genetic differences of local fungal populations due to the result of horizontal gene transfer of transposable elements for the acquisition or loss of pathogenicity [80, 81]. However, horizontal gene transfer has never been studied in fungal GTD pathogens. Many of the Diaporthales are assumed opportunistic pathogens, causing disease only in stressed or weakened hosts or may live endophytically without causing disease [82]. Since these fungi can colonize wounds, the prevalence of these fungi found may be a result of wounds caused by cold injury.

In January of 2019, an atypical polar vortex occurred in the NMW. In Minnesota on January 30, 2019, the temperature dropped to -33°C (-48°C with wind chill) for the Minneapolis-St. Paul area while the lowest temperature recorded in the state was -39°C (-53°C with wind chill) [83]. The polar vortex temperatures were well below the lowest ratings for most of the CHIG varieties in many counties. Winter injury was most apparent on Marquette variety grapevines and in vineyards with little wind protection. Exposure of grapevines to these extreme weather conditions resulted in frost cracks of woody tissues and damage to dormant buds. However, winter injury of grapevines more typically occurs by means of sun exposure. Injury occurs when both direct and snow reflected sunlight warms trunks to above freezing during the day followed by a rapid decrease in temperature to below freezing at night. The sudden drop in temperature ruptures just the outer most layer of phloem cells for mild cases while more serious cases kill cambial cells and damage xylem tissues. When this occurs on trees in the NMW it is often referred to as sunscald. On grapevines this could be considered “winter sunscald”, not to be confused with sunscald of grape berries in the summer. Like extreme cold weather exposure, winter sunscald can also result in both shallow and deep frost cracks depending on severity (Fig 1I–1L). Additionally, winter sunscald of grapevines results in a blackened appearance of the bark on the south to southwest facing side of the vine (Fig 1L). In either case of winter injury often the roots and the lower trunk of vines are protected by the insulating snow covering. Thus, trunk replacement by sucker is a viable and common management strategy in the NMW [84]. Regardless, associated observations of winter injury, vascular discoloration, and identification of fungi suggests our second hypothesis is true and that the polar vortex likely predisposed grapevines to these opportunistic canker pathogens.

Wounds, perhaps from winter injury or mechanical pruning, serve as portals for infection by GTD pathogens under conducive weather conditions such as cool spring or fall rains [32, 85–87]. Fungal spores then colonize and spread through the vascular tissue either by hyphal growth or additional sporulation. Many canker pathogens secrete cell wall degrading enzymes or other compounds to spread laterally through xylem tissues eventually circumnavigating and killing the entire cambium. However, the grapevine host produces tyloses, gels, phenolics, and suberin to compartmentalize the damaged tissues and invading microorganisms [88]. However, restricted balanced production of these defensive structures and compounds is essential. Overproduction of occlusions in response to pathogenic infections can lead to extensive hydraulic failure resulting in external foliar symptoms and often vine death [89, 90]. In cross sections the defense response of the grapevine host is seen as a continuum of brown-red wood to brown-black necrotic tissue (Fig 1). Lighter vascular discoloration indicates more recent responding tissues and likely the front of pathogen spread. Darker vascular discoloration indicates long responding tissues and the probable point of pathogen entry [91]. Alternatively, primarily pectinolytic active pathogens degrade gels in xylem vessels and spread longitudinally by spores through the small spaces between tyloses partially occluding xylem conduits [92]. Longitudinal spread of these pathogens is seen in cross section by the host defense response as black spotting and black lines (Fig 1F) [16]. Genomes of *Cytospora* spp. and *Diaporthe* spp. reveal these fungi employ an abundance of cell wall degrading enzymes [93, 94].

Xylem vessel anatomy likely influences host resistance, pathogen spread, and environmental resilience. In the Dutch elm disease pathosystem, smaller diameter xylem vessels appeared to confer some level of resistance to the causal fungal agents *Ophiostoma ulmi* and *O*. *novo-ulmi* [95]. Reduced vessel diameter permits a more energetically conserved faster occlusion of tissues adjacent to damaged or infected xylem tissues. Pouzoulet et al. (2014; 2017; 2020) conducted histological and pathogenicity studies comparing a few *V*. *vinifera* grapevine cultivars that had varying susceptibility to GTDs and showed cultivars with smaller diameter xylem vessels may likely confer some resistance [88, 96, 97]. Unfortunately, histological pathogenicity studies of grapevines against vascular pathogens are few and completely lacking for hybrid varieties. However, hybrid varieties as well as traditional cultivars have been studied and show links between vessel anatomy and environmental resilience against freezing and drought conditions, though more research is needed [98, 99]. Interestingly, developmental histological studies of grapevine xylem tissues have shown plasticity of vessel diameter even within a variety or individual based on early season precipitation [100]. Therefore, abundant early irrigation influence vines to develop larger xylem vessels that can allow for vigorously growing higher yielding grapevines but may also render vines more susceptible to biotic vascular pathogens and abiotic environmental stresses. Moreover, environmental stresses, including extreme weather events, will be more frequent due to climate change which may provide more opportunities for some vascular pathogens [101]. However, the effects of climate change are likely dependent on the pathogens, cultivars, and environments in question. For example, intensive drought conditions have shown both positive [102] or negative [103] effects for grapevines suffering from GTDs.

Species of *Phaeoacremonium* are found in many grape growing countries and often associated with GTDs of young vines. *Phaeoacremonium* spp. are often found to spread through wounds, nursery propagation and grafting [104–112], see review by Gramaje et al. (2015) [40]. *Ph*. *fraxinopennsylvanicum* is widespread throughout the world on other hosts and has been found in many other investigations in the Midwestern United States [113] but *Ph*. *minimum* appears to be the most widespread *Phaeoacremonium* spp. throughout grape growing countries.

*Cd*. *luteo-olivacea* has been isolated from many substrates including soils [114], decaying wood [115–117], and grapevines [68] as well as from various grafting tools [118] and pruning shears [119]. *Cd*. *luteo-olivacea* is often referred to as a weak pathogen and this fungus was not recognized as pathogenic on grapevine until extended grapevine inoculation studies were conducted (see Table 3). Interestingly, *Cadophora* was the only genus in this study that showed some differences in isolation frequency in comparison with four tested criteria of hypothesis three. No significant differences were observed for any fungal genera isolated compared to variety berry color (S1 Fig). Some researchers have indicated suspicions that red cultivars are more susceptible to GTD pathogens, although much more research is needed [120]. Some significant differences were observed for a few genera compared to sample variety (S2 Fig) or sample county origin (S3 Fig). However, the residuals were only slight for the variety and county origin comparisons and more research is needed to be sure of these correlations. Yet, *Cadophora* spp. were found to be significantly less isolated from cordon sample sections and significantly more isolated from woody sections of trunk, roots, and suckers (S4 Fig). Increased isolation of *Cadophora* spp. from the more central main trunk of the vine may be an indication that infection occurs from the soil or possibly the infection was acquired prior to planting. Additional research of vineyard soils and nursery stock materials would better elucidate the origin of *Cadophora* spp. in NMW grapevines. Additionally, *Ilyonectria liriodendri*, isolated just once in this study, is another weak pathogen often associated with GTDs of roots and often found in nurseries [121, 122].

Fungi considered nonpathogenic to grapevines according to TrunkDiseaseID.org [47] included *Penicillium*, *Alternaria*, *Didymella*, *Epicoccum*, and *Paraconiothyrium* which were some of the more frequently isolated genera in this study. However, few GTD pathogenicity studies have tested Pleosporales fungi. *Paraconiothyrium* spp. have been demonstrated to be pathogenic on fruit trees and other woody species [105] and potentially pathogenic on grapevines [59]. In the NMW, *Paraconiothyrium* spp. could be a potential pathogen. This fungus was recently found associated with the emerald ash borer and found to cause small cankers on healthy ash trees [113, 123]. Further investigation of the pathogenesis of Pleosporales may prove interesting considering the persistence in isolation and sequencing of these fungi.

Basidiomycota have been also found to play a role in GTDs [124–126]. Often the presence of these wood decay fungi are found mainly in older vines following the colonization of faster growing host detoxifying pioneering ascomycota [16]. In many parts of the grape growing regions of the world, the primary Basidiomycota associated with GTDs are *Fomitporia mediterriana* [127] and *Stereum hirsutum* [128]. However, in our study no species of *Fomitporia* were isolated but a different species of *Stereum* was one of the more frequently isolated Basidiomycota. Additionally, *Trametes versicolor* and *Cerrena unicolor* were also isolated. These fungi are commonly found on forest and shade trees locally (personal observations). Fruiting bodies of these fungi have been found on trunks of grapevines that had advanced stages of GTD symptoms. These Basidiomycota have not been tested for pathogenicity on grapevines but *Trametes versicolor* causes cankers and decay on fruit trees [129] and *Cerrena unicolor* is well characterized on hardwoods where it is an aggressive canker rot pathogen [130].

GTD fungi in the Xylariales, Botryosphaeriales, and Phaeomoniellales are of concern in many grape growing countries and this includes fungi in the families Diatrypaceae, Botryosphaeriaceae, and Phaeomoniellaceae [73]. In our study, only 10 Diatrypaceae, and 10 Botryosphaeriaceae isolates were identified. No Phaeomoniellaceae isolates were identified. No species of *Eutypella* or *Eutypa* (both Diatrypaceae), common in some other grape growing regions, were isolated. However, a few isolates not included in analysis closely matched with *Eutypa* but with less than 97% and may prove to be new species following additional detailed taxonomic studies (See S1 Table). *Pestalotiopsis* spp., *Neopestalotiopsis* spp., *Diatrypella verruciformis*, and *Diatrype stigma* of the Xylariales as well as *Diplodia seriata*, *Botryosphaeria dothidea*, *Diplodia corticola*, and *Phaeobotryon negundinis* of the Botryosphaeriales represented a minority of the GTD pathogens isolated in our study (Table 3).

At the start of our research project, many grape growers expressed concerns about GTDs in their young vineyards. There was also considerable concern about Botryosphaeriaceae GTD often called “Bot-rot” or otherwise known as Bot canker. Grape growers in the NMW regularly associate any wedge-shaped discoloration of cross-sections of grapevines as Bot-rot. Based on this survey, Botryosphaeriaceae GTD is rare in the NMW. Confusion and concerns of growers, viticultural professionals, and even fellow researchers is understandable given the complexity of GTDs in addition to the many various names used in an attempt to sub-categorize GTDs. Many of these sub-categorized GTDs have been associated with irregular generalized symptoms of grapevines influenced by the cultivar or variety, climate, and environmental conditions [32, 118, 131]. Moreover, many GTD designations rapidly become obsolete with each taxonomic recategorization of fungal species. Fungal taxonomy will likely continue to change as more genetic information is gathered into databases and mycologist strive to dissolve the two-name system for fungi [132].

The isolation frequency differences of these typically important GTD groups in other grape growing regions is especially notable. Such differences could possibly be correlated with the different climate of the NMW as compared to the many other grape growing regions which typically have more seasonally mild, often Mediterranean climates. Several spore trapping studies from various countries have attempted to characterize sporulation events of various GTD pathogens in correlation with varying weather measurements [87, 133–135]. Given the drastically different GTDs composition of the NMW, additional studies using spore trapping would prove insightful to obtain a better understanding of the GTD pathogens in the NMW.

Notably, culturing methods may present bias as some faster growing fungal species such as those of *Cytospora*, *Diaporthe*, *Diplodia*, and *Botryosphaeria* may outgrow slower growing species such as those of *Phaeoacremonium*, *Phaeomoniella*, and many basidiomycota. This bias of culture-based studies would benefit being paired with modern metagenomic techniques to characterize all potential microorganismal species present in a substrate. However, many metagenomic techniques also present bias such as detection of non-viable organisms or unintended preferential over-identification. Thus, metagenomic techniques also benefit being paired with classical culture-based techniques. Additionally, inclusion of culture-based methods allows for the curation of live microbial collections for use in future pathogenicity or characterization studies. Therefore, future studies which use both classical culturing and metagenomic techniques would better elucidate the NMW GTD complex. This combination of classical and modern techniques has been demonstrated effective in recent local studies of Heterobasidion Root Rot [136]. Regardless, in the study we present here the sample coverage curve (Fig 3) was observed to have reached a plateau providing confidence all major fungal species were identified in this study. Moreover, the use of three types of media allowed for frequent isolation of slow growing fungal species such as those of *Phaeoacremonium* as well as infrequent isolation of fast growing fungal species such as those of the Botryosphaeriales. Therefore, confidence is assured that the isolation frequency of these fungi identified is representative for grapevines of the NMW.

In this study we revealed a large diversity of fungal species associated with cold-hardy hybrid grapevines in the NMW. A handful of these isolates (those with less than 97% sequence match provided in S1 Table) could potentially be revealed as new species following additional detailed taxonomic studies. However, the majority of fungal species we identified show Diaporthales predominate GTDs in the NMW. Diaporthales GTD species, *Cytospora* and *Diaporthe*, are generally opportunistic fungi and largely spread to new hosts within short distances by asexual conidia via rain splash or contaminated tools. Basic understanding of these opportunistic pathogens lifecycles emphasizes the benefit growers would gain from more intentional phytosanitary practices such as prompt removal and destruction of pruning debris as well as the regular sanitization of tools. Pruning debris and diseased canes left unpruned have recently been shown to be a major source of *Diaporthe* GTD Inoculum [135]. Our current recommendation for grape growers in the NMW is to prune in the dormant winter season during a period of cold and dry weather. Recommendations on pruning timing could be fine-tuned by epidemiological spore trapping studies in NMW vineyards and may possibly allow for some degree of GTDs forecasting. Vineyard spore trapping could also provide the opportunity for broader biosurveillance of invasive pathogenic microbial species of forest, shade, and orchard trees.

Knowing the prevalence of GTDs in the NMW provides insight for the development of improved management practices. Similar studies of GTD pathogens spread from nurseries of cold-hardy grapevine hybrid varieties would also provide insight to improved propagation practices as well as yield less stressed, higher quality, and more vigorous growing nursery stock plants for growers. Assessment of these hybrid varieties against a panel of GTD pathogens may reveal novel evidence of resistance or susceptibility that would be useful for grape breeders. The development of cost-effective rapid molecular assays for the most prevalent GTDs in the NMW would be a useful tool to measure the effectiveness of practices or the variability of variety susceptibility to GTDs.

## Supporting information

S1 Table Isolates with less than 97% homology match. Taxonomic rankings from order to species are denoted followed by isolation frequency in parenthesis. The isolation frequency is the count of samples each taxa was isolated from a possible 172 samples. Isolates deposited to GenBank are listed for each species along with the sample variety and county origin of that isolate. Pathogenicity studies conducted for each species are listed in brackets following species. See references for complete citations. Highlighted species have associated pathogenicity studies.
(DOCX)

S1 Fig Mosaic plot of genus level taxa isolated from sample varieties. (TIF)

S2 Fig Mosaic plot of genus level taxa isolated from sample variety berry color. (TIF)

S3 Fig Mosaic plot of genus level taxa isolated from sample section types. (TIF)

S4 Fig Mosaic plot of genus level taxa isolated from counties. (TIF)
