# Supplementary material for: Grapevine trunk diseases of cold-hardy varieties grown in Northern Midwest vineyards coincide with canker fungi and winter injury
Source: PLoS One. 2022 Jun 3;17(6):e0269555. doi: 10.1371/journal.pone.0269555 (PMC9165834; doi:10.1371/journal.pone.0269555)
Supplement: S1 Table — Taxonomic rankings from order to species are denoted followed by isolation frequency in parenthesis. The isolation frequency is the count of samples each taxa was isolated from a possible 172 samples. Isolates deposited to GenBank are listed for each species along with the sample variety and county origin of that isolate. Pathogenicity studies conducted for each species are listed in brackets following species. See references for complete citations. Highlighted species have associated pathogenicity studies. (DOCX) [file pone.0269555.s001.docx]

| **Order (n)** | **Family (n)** | **Species (n)** | **Isolate** | **Host Variety** | **Origin** | **GenBank** |
| --- | --- | --- | --- | --- | --- | --- |
| Helotiales (3) | undefined Helotiales family (3) | *Cadophora fastigiata* (2) | GI-237 | St. Pepin | Goodhue, MN | OM307693 |
|  |  |  | GI-501 | La Crescent | Walworth, WI | OM307694 |
|  |  | *Cadophora malorum* (1) | GI-473 | La Crescent | Winona, MN | OM307695 |
| Hypocreales (2) | undefined Hypocreales family (1) | *Cephalosporium gramineum* (1) | GI-380 | Frontenac Blanc | Crow Wing, MN | OM307696 |
|  | Stachybotryaceae (1) | *Myrothecium penicilloides* (1) | GI-862 | Marquette | Goodhue, MN | OM307697 |
| Pleosporales (12) | Phaeosphaeriaceae (11) | *Neosetophoma cerealis* (9) | GI-513 | Brianna | Le Sueur, MN | OM307698 |
|  |  |  | GI-854 | Valiant | Douglas, MN | OM307699 |
|  |  |  | GI-855 | Edelweiss | Trempealeau, WI | OM307700 |
|  |  |  | GI-283 | La Crescent | Goodhue, MN | OM307701 |
|  |  |  | GI-288 | Marquette | Jackson, MN | OM307702 |
|  |  |  | GI-295 | Brianna | Jackson, MN | OM307703 |
|  |  |  | GI-294 | Brianna | Jackson, MN | OM307704 |
|  |  |  | GI-39 | Marquette | Meeker, MN | OM307705 |
|  |  |  | GI-478 | Frontenac Blanc | Crow Wing, MN | OM307706 |
|  |  | *Neosetophoma rosigena* (2) | GI-92 | Marquette | Meeker, MN | OM307707 |
|  |  |  | GI-139 | La Crescent | Goodhue, MN | OM307708 |
|  | Didymosphaeriaceae (1) | *Paraphaeosphaeria sporulosa* (1) | GI-203 | La Crescent | Goodhue, MN | OM307709 |
| Xylariales (13) | Sporocadaceae (8) | *Seiridium marginatum* (5) | GI-840 | Frontenac Blanc | Fillmore, MN | OM307710 |
|  |  |  | GI-411 | Frontenac | Douglas, MN | OM307711 |
|  |  |  | GI-324 | Marquette | Goodhue, MN | OM307712 |
|  |  |  | GI-410 | Marquette | Blue Earth, MN | OM307713 |
|  |  |  | GI-349 | La Crescent | Fillmore, MN | OM307714 |
|  |  | *Seiridium podocarpi* (1) | GI-430 | Frontenac | Douglas, MN | OM307715 |
|  |  | *Seiridium unicorne* (1) | GI-185 | St. Pepin | Pine, MN | OM307716 |
|  | Diatrypaceae (4) | *Eutypa lata* (3) [38,56,62,63,135,136] | GI-439 | Frontenac | Winona, MN | OM307717 |
|  |  |  | GI-250 | La Crescent | Fillmore, MN | OM307718 |
|  |  |  | GI-241 | Marechal Foch | Goodhue, MN | OM307719 |
|  | Hypoxylaceae (1) | *Hypoxylon rubiginosum* (1) | GI-154 | La Crescent | Carver, MN | OM307720 |
| Russulales (7) | Stereaceae (3) | *Stereum gausapatum* (3) | GI-436 | Prairie Star | Winona, MN | OM307721 |
|  |  |  | GI-835 | Prairie Star | Winona, MN | OM307722 |
|  |  |  | GI-67 | Marquette | Carver, MN | OM307723 |
|  |  | *Stereum hirsutum* (1) [3] | GI-197 | Marquette | Carver, MN | OM307724 |
| Tremellales (1) | Bulleribasidiaceae (1) | *Vishniacozyma globispora* (1) | GI-113 | unknown | Carver, MN | OM307725 |
| Mucorales (1) | Cunninghamellaceae (1) | *Gongronella butleri* (1) | GI-188 | Marquette | Goodhue, MN | OM307726 |
