# Supplementary figures and images for: Grapevine trunk diseases of cold-hardy varieties grown in Northern Midwest vineyards coincide with canker fungi and winter injury
Source: PLoS One. 2022 Jun 3;17(6):e0269555. doi: 10.1371/journal.pone.0269555 (PMC9165834; doi:10.1371/journal.pone.0269555)

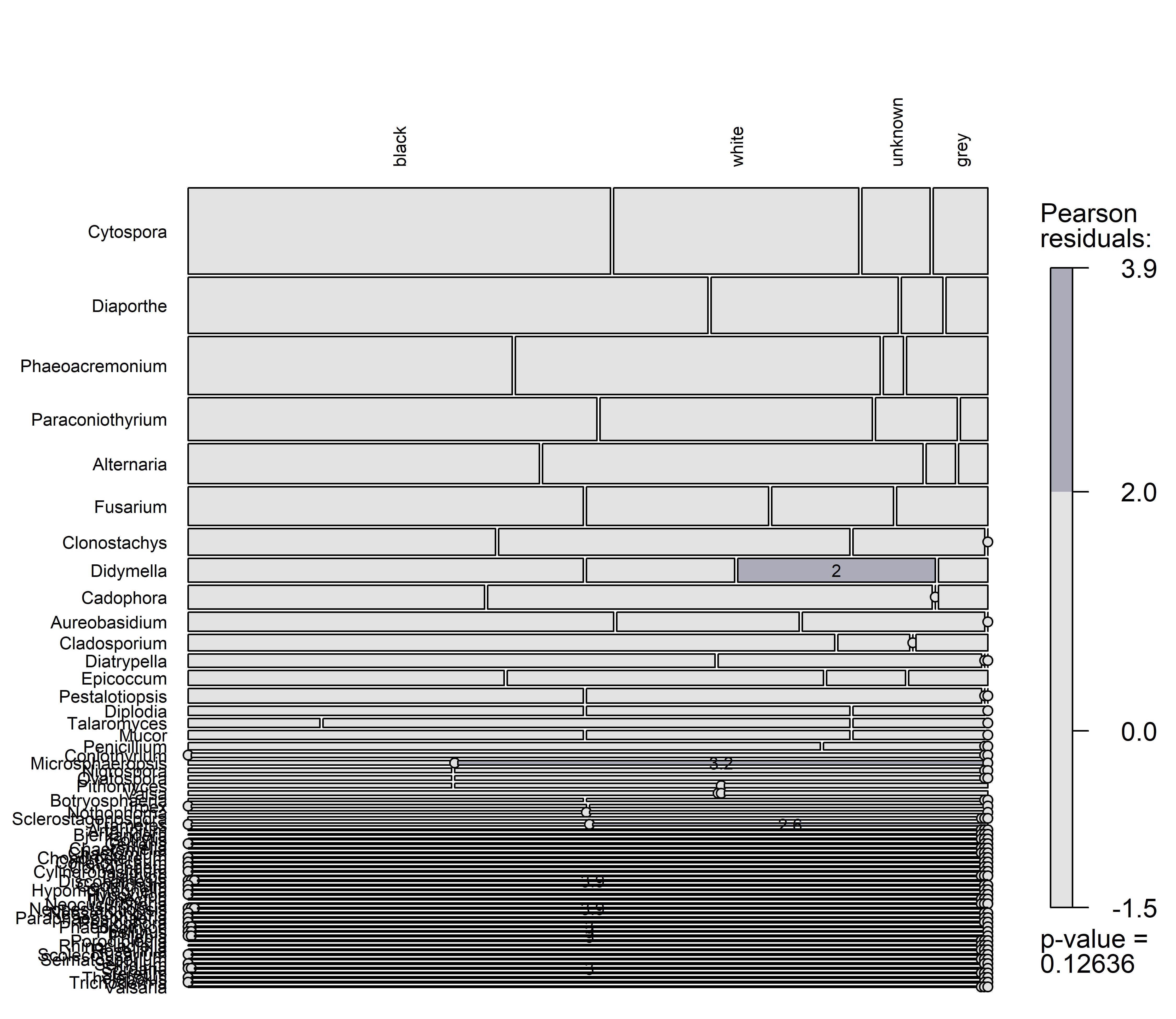

Supplement: S1 Fig — (TIF) [file pone.0269555.s002.tif]

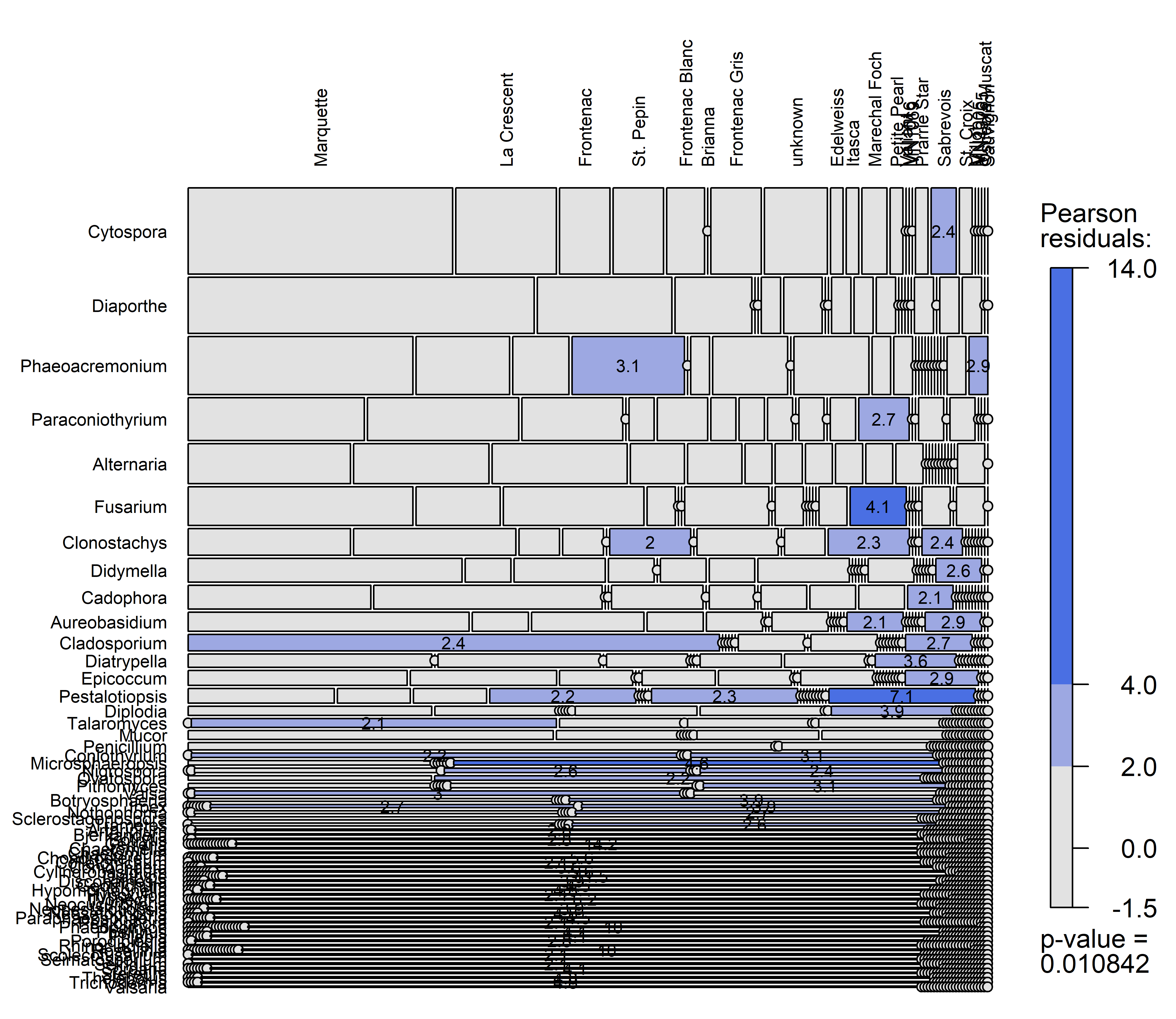

Supplement: S2 Fig — (TIF) [file pone.0269555.s003.tif]

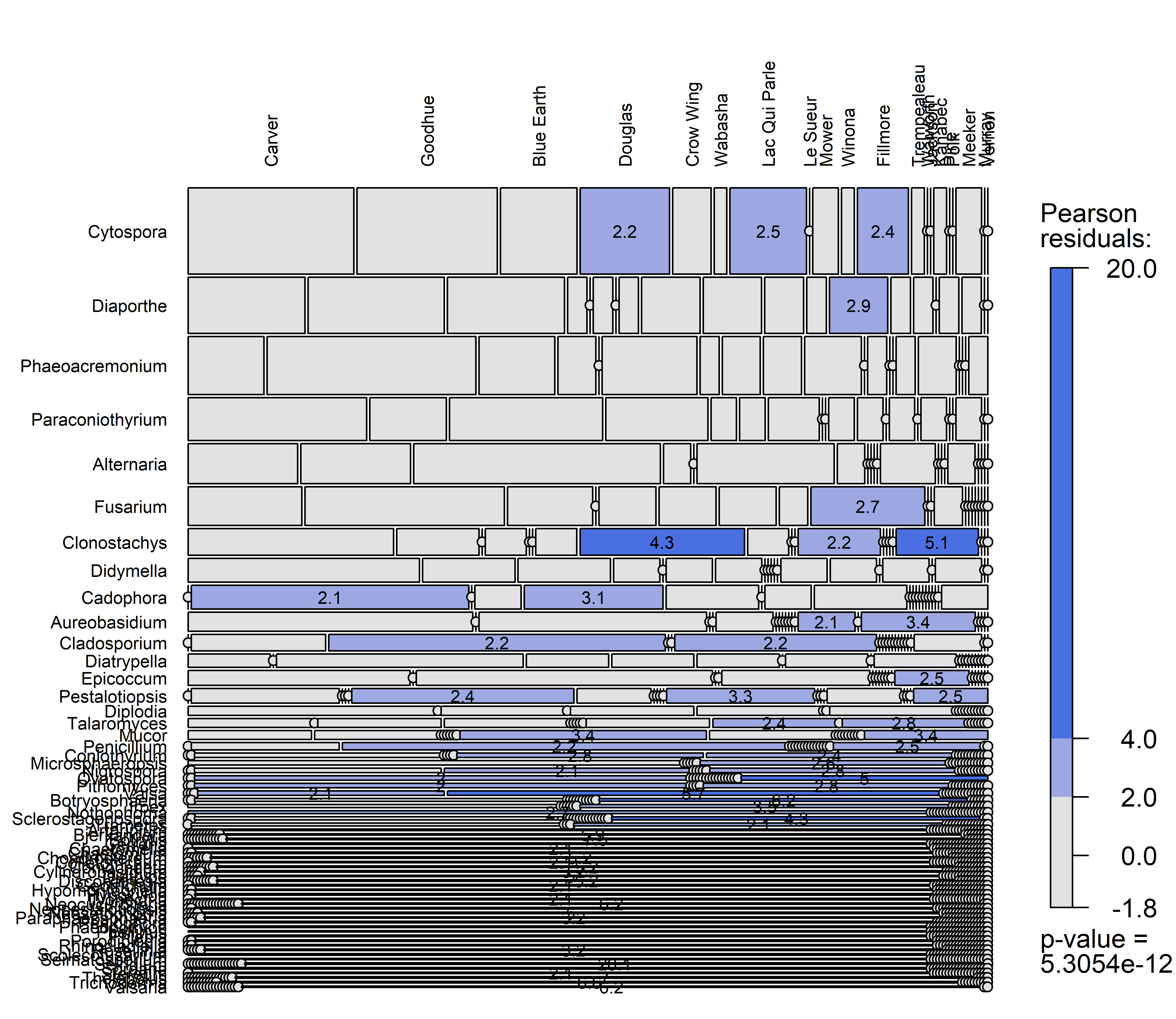

Supplement: S3 Fig — (TIF) [file pone.0269555.s004.tif]

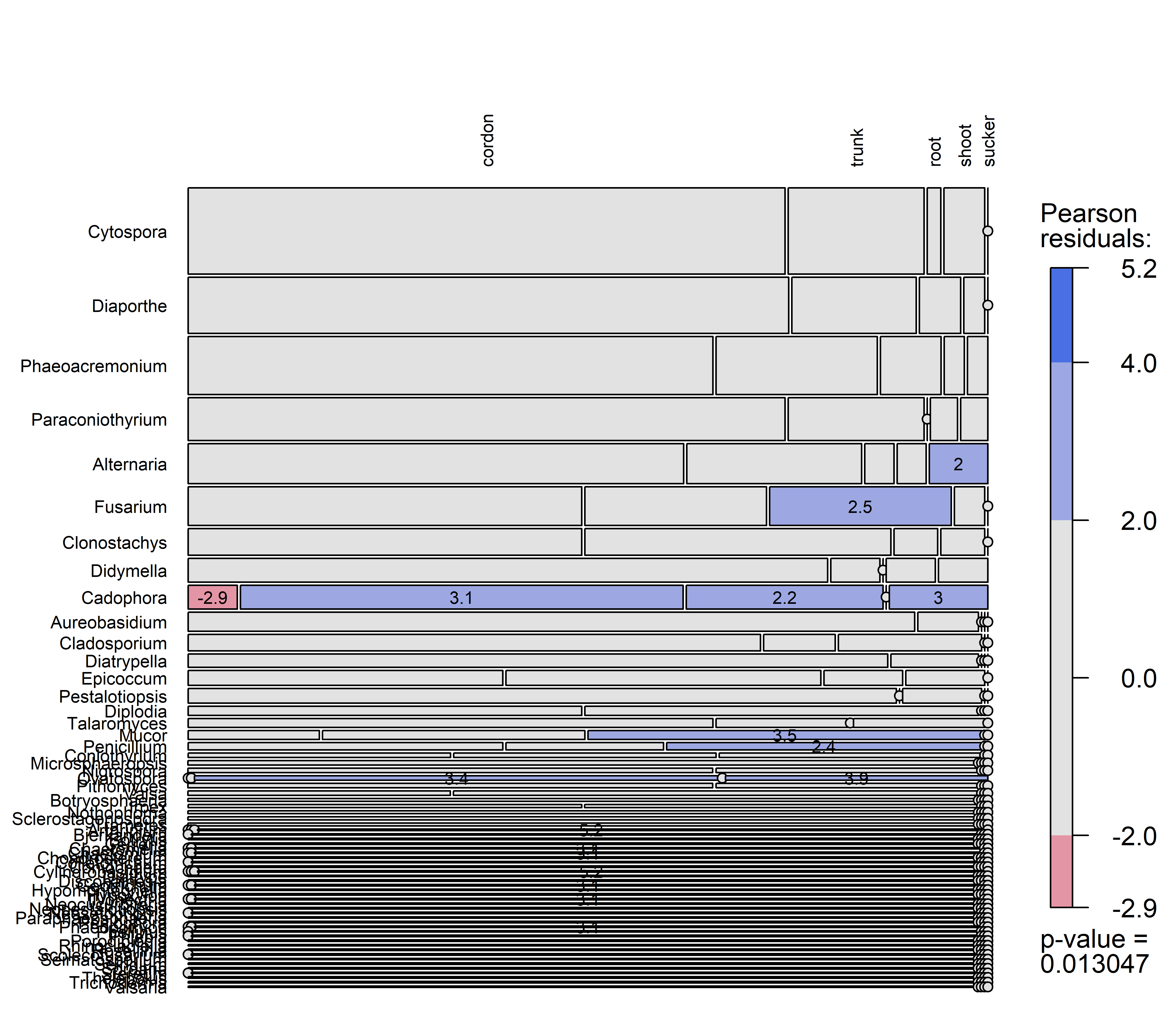

Supplement: S4 Fig — (TIF) [file pone.0269555.s005.tif]
